# Cases of Longevity

**Published:** 1884-02

**Authors:** 


### CASES OF LONGEVITY.

The distinguished Galen, the versatile and accomplished medical writer of the second century, died at the reputed age of 140 years; but this is disputed and the time fixed at eighty years. Abraham Bogart died in Tennessee at the age of 118 years, and Francis Age, of Pennsylvania, significantly served out six score and fourteen years. Stephen Goodale died not long ago in York (Me.) Poor-House at 118. Joseph Crele died at Caledonia, Wis., in January, 1865, aged 140, and about the same time Antonine Sauve died in France at 130; his father fought Marlborough, May 3, 1706. Longevity has been ascribed to various conditions and causes, regularity of habit and morality conducing largely to it; but these explanations are contradicted in the lives of persons who have reached far beyond the centennial period. Long life is not dependent upon habit, nor employment, so far as the data before us throws light upon the subject. It has been contended that intellectual pursuits shorten the term of life, but this is refuted by numerous examples. Rev. William Davies, a vicar, reached 105, although for thirty-five years before his death his exercise led him only from one room of his house to another, aud he ate and drank to excess, preserving robust health to the end. Daniel McCarthy, of Kerry, Ireland, dissipated through the last seventy years of his life, dying at 111. Geo. Kirton, of Yorkshire, drank more than frequently, and expired at 120. Personal cleanliness, though a great merit, found an antagonist in Lady Lewson, a London widow, who eschewed all pretensions to cleanliness and died at 106. She rejected water, because it gave her a cold, and polished her face and neck with hog's lard. Elizabeth Duriex, of Savoy, notoriously filthy, survived to 119. Longevity does not depend on health, as many can certify who have themselves attained any considerable age. The stoutest and most muscular representatives of the race die early. Lewis Carnaro was informed by his doctors at thirty-five that he could not last two years, and yet he went through sixty-five more ere he flitted this mundame sphere. Galen had no constitution and shook like a reed in the wind. Despite these cases abstemiousness, physical exercise and virtuous conduct do contribute to swell the number of our years; there are exceptions to all rules. Sir Thomas Browne believed that some persons had an aptitude for long duration. There can be no doubt that a disposition to live will prolong the tenure. That longevity is an inheritance descending from ancestry is a well established fact; that it originally came from habits of life cannot be gainsaid; and that it can be cultivated, acquired, is attested by history and experience. Our sweetest poet, William Cullen Bryant, when asked how he accounted for his vigor, then over eighty years of age, replied : "It is all summed up in one word—moderation." Frugal living, abstenance from all that injures, denial of stimulants that will not provoke unnatural heart throbs and wasted vitality. Swift thought men of head-and-heart-worry died young ; yet Wordsworth, who agreed with him, saw eighty summers ; and the poet Rogers, who had no sunshine in his soul, dwelt through the shadows of ninety-two years. Mental toil does not diminish, perhaps extends, probation. Lord Brougham, extremely laborious, wrought eighty-nine years ; Lord Lyndhurst, ninety-one, and Epimenides, of the seven wise men, 154, it is related. But the number of brain workers who passed three and four score is too large for mention in this space. Fontenelle outlived a century, and when asked as he was dying if he felt pain said, "I only feel the difficulty of existing." (Singularly, an exactly similar sentiment was uttered by the dying Garfield.) Again, a friend of ninety who approached him said, "Death, I think, has forgotten us." "Hush !" whispered Fontenelle, "he may overhear us." Bacon declared that literary life produced longevity. Plato died in his eighty-first year while writing. Isocrates wrote his "Panathenaican" in his ninety-fourth year and lived five years more. Gorgias, the Leontine, completed 118 years, laboring to the end of his life. Cato learned Greek at eighty, and wrote his seventh book of "Antiquities" in his eighty-fourth year. Cicero found it recorded that Arganthonious reigned at Gades, on the island of Cadiz, and lived 120.

